# Myopia control: short-term effect of 0.01% atropine vs. defocus incorporated multiple segment lenses—a retrospective study in European children

**DOI:** 10.1007/s10792-023-02788-x

**Published:** 2023-07-07

**Authors:** Sandra Guimarães, Patrícia Barros da Silva, Bárbara Oliveiros, Eduardo Silva

**Affiliations:** 1 Instituto de Investigação, Inovação e Desenvolvimento da Universidade Fernando Pessoa (FP-I3ID), Gondomar, Porto, Portugal; 2 Hospital-Escola da Universidade Fernando Pessoa (HE-UFP), Gondomar, Porto, Portugal; 3 Escola Superior de Saúde da Universidade Fernando Pessoa (ESS-UFP), Gondomar, Porto, Portugal; 4 CRI – OftaPed, Hospital Dona Estefânia, Centro Hospitalar Universitário de Lisboa Central, Lisbon, Portugal; 5 Coimbra Institute for Clinical and Biomedical Research, Faculty of Medicine (iCBR-FMUC), University of Coimbra, Coimbra, Portugal; 6 Laboratory of Biostatistics and Medical Informatics, Faculty of Medicine (LBIM, FMUC), University of Coimbra, Coimbra, Portugal; 7 Department of Ophthalmology, Centro Hospitalar Universitário de Lisboa Central, Lisbon, Portugal

**Keywords:** Myopia control, Atropine, Pediatric, Multiple segment spectacle lenses, Axial length

## Abstract

**Purpose:**

To compare 0.01% atropine with DIMS spectacle lenses in the prevention of myopia progression in European children.

**Methods:**

This was a retrospective study including data from pediatric European patients with myopia. From November 2021 to March 2022, only 0.01% atropine was prescribed because DIMS lenses were still not available in Portugal. From March to October 2022, only DIMS spectacle lenses were prescribed due to patients’ parents’ preference. Myopia progression endpoints were axial length (AL) and spherical equivalent (SE) differences between before and 6 months after treatment. AL and SE evolution were compared using a general linear model with repeated measures.

**Results:**

The study included 98 eyes from 50 patients: 47 in the atropine group and 51 in the DIMS group. There were no statistically significant differences between groups in terms of initial AL, initial SE, sex or age. The mean AL elongation at 6 months was 0.057 mm in the atropine group (SD = 0.118) and 0.002 mm (SD = 0.077) in the DIMS group. SE progression was − 0.098 (SD = 0.232) D in the atropine group and − 0.039 (SD = 0.105) D in the DIMS group. AL elongation was significantly lower in the DIMS lens group (*p* = 0.038, partial Eta^2^ = 0.045). There was no difference in SE progression between groups (*p* = 0.302, partial Eta^2^ = 0.011).

**Conclusion:**

Comparison between 0.01% atropine eyedrops and DIMS spectacle lenses for slowing the progression of myopia favored DIMS lenses in terms of AL elongation in a short-term follow-up. There was no difference in terms of SE between groups.

## Introduction

In the last decade, the prevalence of myopia has been growing significantly, and numbers are evolving to a real pandemic. In 2010, in Europe alone, 227.2 million individuals were myopes [1]. A systematic review pointed out that by 2050, there will be 4758 million myopes in the world (49.8% of the world’s population) [2]. Uncorrected myopia is responsible for distance vision impairment. High myopia is associated with an increased risk of retinal detachment, myopic macular degeneration, myopic choroidal neovascularization and glaucoma [3–5]. An increase in the prevalence of myopia implies other related costs, a greater risk of comorbidities and loss of quality of life [6].

Due to its burden, myopia progression control has become a research focus in the last decade. From school-based outdoor promotion programs to repeated low-level red-light treatments, many options have been proposed [7, 8]. Within the different studied methods, atropine drops and defocus incorporated multiple segments (DIMS) spectacle lenses are among the most effective [9, 10].

Topical atropine has been tried at different concentrations for many years. The initial trials used higher atropine concentrations (0.5–1%) and reported reduced progression in myopic refraction in treated eyes compared to placebo [11, 12]. However, higher concentrations were associated with more disturbing side effects and less treatment compliance. More recent studies revealed good clinical outcomes with lower atropine concentrations (0.01–0.1%), with fewer adverse effects and better compliance. In a 5-year trial comparing different atropine concentrations, Chia et al. concluded that atropine 0.01% eyedrops were more effective in slowing myopia progression with less visual side effects than higher doses [13].

In parallel to pharmacological interventions, DIMS spectacle lenses are an emerging option showing promising results. Siu Yin Lam published a randomized clinical trial that showed significant slow-down of myopia progression and axial elongation in myopic children wearing DIMS compared to single vision spectacle lenses [14]. The clinical outcomes were confirmed at the 3- and 6-year follow-ups [15, 16].

Among the different emerging options, there are still no definite treatment guidelines regarding myopia progression. There is only one publication comparing low-dose atropine and DIMS lenses [17]. Moreover, most clinical studies report results from Asian populations, in which myopia is more prevalent and might not reflect the results in European populations. In a recent network meta-analysis, Downie et al. tried to assess the comparative efficacy of optical, pharmacological and environmental interventions for slowing myopia progression in children. They concluded that there is enough evidence that these interventions may slow refractive change and reduce axial elongation but there is still lack of combination studies [18].

The aim of this study is to compare atropine 0.01% with DIMS Spectacle Lenses in slowing the progression of myopia in children in a European descendent population.

## Materials and methods

### Study design and subjects

This is a retrospective study that includes two groups of pediatric patients with myopia evaluated at private practice from November 2021 to January 2023. From November 2021 to March 2022, only 0.01% atropine was prescribed because DIMS lenses were still not available in Portugal. From March 2022 to October 2022, only DIMS spectacle lenses were prescribed due to patients’ parents’ preference.

*Inclusion criteria* treatment-naïve patients with a diagnosis of myopia with cycloplegic spherical equivalent refraction (SE) at least − 0.75; age between 4 and 17 years old.

*Exclusion criteria* patients who had already undergone any type of myopia prevention treatment; ophthalmic disorders such as glaucoma, cataract, keratopathy, strabismus, and amblyopia; use of cholinergic or anticholinergic drugs such as atropine, pirenzepine, and pilocarpine within the last 1 month.

Patients were evaluated before starting myopia prevention treatment and at 6 months of treatment. Data from both eyes were collected in every patient.

The atropine group was prescribed 0.01% atropine eyedrops to be applied once per day before bedtime in both eyes. In the DIMS spectacle lens group, subjects were required to wear DIMS spectacle lenses all day every day. Costs were supported by patients in both groups.

The primary outcome was myopia progression through spherical equivalent (SE) and axial length (AL) increase.

### Informed consent

Consent was obtained from parents and children. Before starting the treatment, information about the benefits and risks of using both atropine and DIMS spectacle lenses was explained to the parents. We also provided a simple and easy-to-understand explanation to the children. This study was performed in line with the principles of the Declaration of Helsinki. It was approved by the ethics committee of Hospital School of University Fernando Pessoa (HE-UFP).

### Methods

We retrospectively collected data on sex, age, objective refraction and axial length.

The axial length (AL) before and 6 months after treatment was obtained using MYAH® (Topcon Healthcare Inc., Tokyo, Japan) and objective refraction with Auto Refkeratometer KW 2000® (Kowa Company, Ltd., Aichi, Japan). We applied cyclopentolate hydrochloride 1% 3 times within 10 min and waited at least 40 min before complete cycloplegic autorefraction. We always retrieved 3 values for autorefraction. Whenever these values were not equal, one additional drop of cyclopentolate hydrochloride 1% was instilled and cycloplegic refraction was repeated until the values were not different, to ensure that cycloplegia was complete. The same examiner performed all the evaluations.

MYAH® (Topcon Healthcare Inc., Tokyo, Japan) was chosen due to its normative growth curves created using the extensive axial length dataset collected by Erasmus University (Rotterdam, NL) [19]. The device was used according to the user’s manual.

### Statistical analysis

Statistical analyses were produced using SPSS Statistics 28.0 (IBM Corp., Armonk, NY). All statistical tests were performed by a 2-sided test. A significance level of 5% was considered.

Objective refraction was converted to spherical equivalent calculated as spherical power plus half of the cylinder power. The results obtained were expressed as mean and standard deviation. Student’s T-test was used to compare mean patient age, initial AL and SE, and chi-square test was used to compare sex between groups. For this, we considered the patient as the unit of analysis.

AL and SE evolution was compared in the two groups using a general linear model (GLM) with repeated measures. We used both the right and left eyes in our analysis. To overcome this limitation, we classified each eye as best or worst eye and used this variable as covariate in the linear model. The eye with a greater AL was chosen as worst eye. Whenever only one eye was analyzed, it was considered the worst eye.

## Results

### Baseline characteristics

The study included a total of 98 eyes from 50 patients: 47 in the atropine 0.01% group and 51 in the DIMS group. The odd number was caused by two patients in which only 1 eye was included: one with a congenital cataract on the other eye and the other only had myopia in one eye. All patients who started the study did the 6 months review. The mean patient age was 10.64 (SD = 2.49). All the patients were of Caucasian background. For baseline characteristics analysis, we only included the worst eye for each patient, with a total of 50 patients (24 in the atropine group and 26 in the DIMS lens group). Baseline demographic and clinical characteristics are described in Table 1. There were no significant differences between groups in terms of initial AL, initial SE, sex or age.

**Table 1:** Baseline demographic and clinical Characteristics

Characteristic	Total	Atropine group	DIMS group	*p* value
Age, years (SD)	10.66 (2.52)	10.10 (2.35)	11.17 (2.61)	0.136
Sex, feminine (%)	25 (50)	11 (46)	14 (54)	0.389
Baseline axial length, mm (SD)	24.26 (0.82)	24.31 (0.84)	24.21 (0.83)	0.664
Baseline spherical equivalent, diopters (SD)	− 2.23 (1.31)	− 2.44 (1.28)	− 2.04 (0.83)	0.293

### Changes in spherical equivalent and axial length

At 6 months, the mean axial length elongation at 6 months was 0.057 mm in the atropine group (SD = 0.118) and 0.002 mm (SD = 0.077) in the DIMS group. Spherical equivalent progression was − 0.098 (SD = 0.232) D in the atropine group and  − 0.039 (SD = 0.105) D in the DIMS group.

**Fig. 1:**
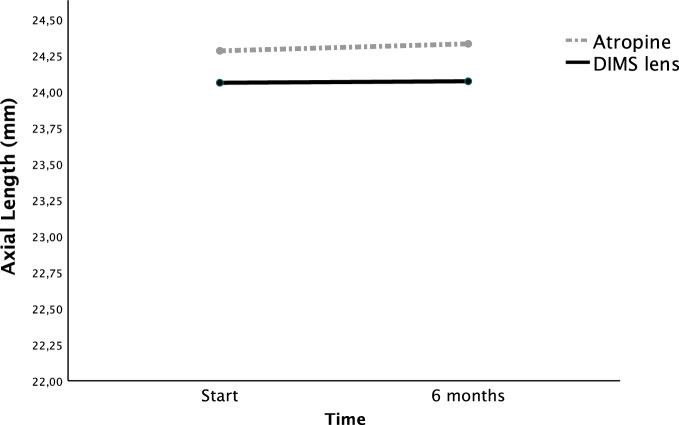
Axial length (AL) progression. AL elongation was significantly lower in the DIMS lens group (*p* = 0.038, partial Eta^2^ = 0.045)

**Fig. 2:**
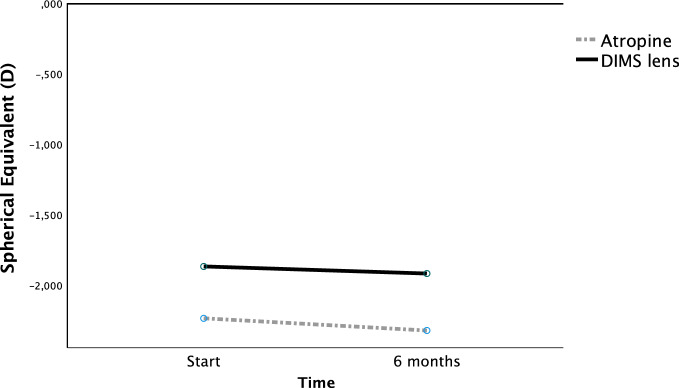
Spherical equivalent (SE) progression. There was no difference in SE progression between groups (*p* = 0.302, partial Eta^2^ = 0.011)

We performed a GLM with repeated measures for AL and SE, admitting age and best/worst eye as covariates. Figure 1 represents the GLM for AL progression at 6 months. Figure 2 represents the GLM for SE progression at 6 months. AL elongation was significantly lower in the DIMS lens group (*p* = 0.038, partial Eta^2^ = 0.045). There was no statistically significant difference n SE progression between groups (*p* = 0.302, partial Eta^2^ = 0.011). In both AL and SE GLM time and age were statistically significant for AL and SE variation at the two time points. The best/worst eye did not influence AL or SE progression (*p* = 0.355, partial Eta^2^ = 0.009 for AL and *p* = 0.780, partial Eta^2^ = 0.001 for SE). Table 2 shows the AL and SE results in the GLM for time, age and treatment.

**Table 2:** General linear model for axial length and spherical equivalent, with age and best/worst eye as covariates

	Effect	Partial Eta^2^	*p* value
Axial length	Time	0.213	< 0.001
	Time × age	0.198	< 0.001
	Time × best/worst eye	0.009	0.355
	Time × treatment	0.045	0.038
Spherical equivalent	Time	0.125	< 0.001
	Time × age	0.110	< 0.001
	Time × best/worst eye	0.001	0.780
	Time × treatment	0.011	0.302

## Discussion and conclusion

In a group of children aged between 4 and 17 years old with myopia, nightly use of low-dose atropine eyedrops compared with DIMS spectacle lenses for slowing the progression of myopia showed a statistically significant difference in terms of axial length elongation, favoring DIMS lenses. However, there was no statistically significant difference between groups in SE variation in a short-term follow-up.

An extension of the follow-up period is necessary to evaluate whether the long-term effects maintain this difference between treatments and/or if there might also be a delayed SE difference between groups.

In a recent nonrandomized experimenter-masked prospective controlled observational study, Nucci et al. compared 0.01% atropine eyedrops, DIMS (Hoya® MiyoSmart®) spectacles, combined atropine  + DIMS or single vision spectacle lenses (control group) [17]. In pairwise comparisons at 6 and 12 months the atropine + DIMS group had significantly reduced SE progression compared with the DIMS only and atropine only groups (*p* < 0.001). Regarding the comparison between atropine only and DIMS only at 6 months they did not find statistically significant difference between groups in terms of SE (*p* = 0.53) nor AL (*p* = 0.99). In the methods, the authors say they calculated SE as a median of 3 readings for each measurement, meaning they have obtained different readings for SE in each patient. This might point to incomplete cycloplegia in some patients, which could have compromised the results. In the comparison between 0.01% atropine and DIMS spectacles, the authors found no difference at 6 months (*p* = 0.99) or at 12 months (*p* = 0.82), contradicting our findings concerning AL.

The low-concentration atropine for myopia progression (LAMP) study compared the efficacy 0.05%, 0.025%, and 0.01% atropine for myopia progression prevention in a Chinese population for 3 years. Both at 8 and 4 months 0.01% atropine compared with placebo significantly reduced both SE and AL progression. The results showed 0.05% atropine to be the optimal concentration [20]. It might be possible that the 0.01% atropine concentration widely used is not enough to prevent myopia progression and that the inferiority compared to DIMS lenses that we found could be overcome with a higher concentration, such as 0.025% or 0.05%. A recent systematic review investigating the effectiveness of myopia control interventions by Lanca et al. reported that low-dose atropine 0.01% was not effective in reducing AL progression in two studies at 12 months. Treatment efficacy with low-dose atropine of 0.05% showed good efficacy [21].

The Shenzhen Kindergarten eye study evaluated AL, lens power (LP), corneal curvature and SE; the authors found significant axial elongation with minimal change in refraction in children aged between 3 and 6 years old [22]. They concluded that an increase of 1 mm in AL was only associated with a − 0.45-D change in SE due to the loss of crystalline LP. We found a significant difference in AL between the DIMS lens group and the atropine group. It is possible that the significant difference that we found in AL between treatments was not reflected in SE because of the atropine interference in lens format and LP due to its cycloplegic effect. Another study in Chinese children reported a delay between AL progression and SE change [23]. This could mean that these treatments might not have a simultaneous effect on the two parameters and that only with a longer follow-up it would be possible to detect a significant difference in SE between groups.

### Limitations

One limitation of our study is the inclusion of both right and left eyes for each patient. We tried to correct for this handicap by classifying each eye as the best or worst eye and used this variable as a covariate in our linear model. Our results show that the variable best/worst eye did not influence AL or SE progression.

Being a retrospective study in the real-life setting, we lack randomization. This could bring some bias to our analysis. Despite this, one of the most important factors impacting myopia progression is age, which was not significantly different between groups [24, 25].

Another limitation was the absence of a priori sample size computation in the study design phase. We overcame this constraint computing achieved power in GPower 3.1.9.7 based on the achieved effect size *f* = 0.217 for the within-between interaction in GLM model (two repeated measures in two groups) with a sample size of 98 for which we have obtained a statistical power of 98.9%.

Other factors that we did not consider in our analysis were parental history of myopia, time spent reading, and time spent in outdoor/sports activity, which could also work as covariates in this type of study [26, 27].

### Impact in real life

Both atropine and DIMS spectacle lenses have advantages and disadvantages. The cost of atropine drops and DIMS spectacles depends on the countries’ access. In Australia, the lowest lifetime cost options are anti-myopia spectacles, while in China, low-dose atropine is less expensive [28]. In Portugal, compound low-concentration atropine for 15 days can be priced between 7 and 30€, depending on the pharmacies. It has some practical disadvantages, such as the need for parents and children to cooperate and comply. Moreover, in the Portuguese setting, there is no approved diluted atropine, so parents must buy new pharmacy-compounding drops every fortnight. On the other hand, DIMS spectacle lenses are far more practical because these children are already used to wearing glasses. The difference between price depends on how frequently DIMS lenses are changed and on pharmacies’ price for compound atropine.

Until now, there was no evidence to prefer one treatment over the other, meaning that ophthalmologists would choose the method that best suited their patient needs, both in terms of comfort, compliance, accessibility, and economic factors. The fact that DIMS lenses might be more effective in slowing the progression of myopia in terms of AL compared to atropine 0.01% could mean that there is a reason to prefer this treatment.

### Conclusions

In our study, nightly use of low-dose atropine eyedrops compared with DIMS spectacle lenses for slowing the progression of myopia favored DIMS lenses in terms of AL elongation. There was no difference in terms of SE between groups in a short-term follow-up.

Findings of previous studies have already shown that both low-dose atropine and DIMS spectacle lenses decrease myopia progression [15, 20].

Further research is needed to replicate our findings and to increase the follow-up period to evaluate whether the long-term effects maintain this difference between treatments and/or if there might also be a delayed SE difference between groups.
